# Successful treatment of mediastinal pancreatic pseudocyst and pancreatic pleural effusion with endoscopic pancreatic duct drainage: A case report

**DOI:** 10.1002/deo2.133

**Published:** 2022-06-05

**Authors:** Shunsuke Watanabe, Masao Toki, Komei Kambayashi, Shuichi Kitada, Takeshi Nosaka, Kazushige Ochiai, Koichi Gondo, Junji Shibahara, Tadakazu Hisamatsu

**Affiliations:** ^1^ Department of Gastroenterology and Hepatology Kyorin University School of Medicine Tokyo Japan; ^2^ Department of Pathology Kyorin University School of Medicine Tokyo Japan

**Keywords:** chronic pancreatitis, dyspnea, endoscopic transpapillary pancreatic duct drainage, mediastinal pancreatic pseudocyst, pancreatic pleural effusion

## Abstract

An 81‐year‐old man with chronic pancreatitis was being treated with a protease inhibitor. He developed an acute exacerbation of chronic pancreatitis and dyspnea. Contrast‐enhanced computed tomography showed disruption of the main pancreatic duct, a cystic lesion connecting the mediastinum to the main pancreatic duct, and left pleural effusion. We diagnosed a pancreatic pseudocyst, mediastinal pancreatic pseudocyst, and pancreatic pleural effusion. Endoscopic retrograde pancreatography showed leakage of contrast medium from the pancreatic body; furthermore, a cystic cavity extending to the mediastinum through a pancreatic duct fistula was visualized. An endoscopic transpapillary nasopancreatic drainage tube was placed in the cystic cavity. Computed tomography showed that the mediastinal pseudocyst and pleural effusion had disappeared. Endoscopic transpapillary pancreatic duct drainage may be useful when a connection between the main pancreatic duct and a mediastinal pseudocyst is confirmed by imaging.

## INTRODUCTION

Pancreatic pseudocysts are a common complication of acute or chronic pancreatitis. Most are found around the pancreas or in the retroperitoneal space; in rare cases, however, they have been reported to extend into the mediastinum.^1^ This differs from pleural effusion, which accumulates by the direct inflammatory spread of pancreatitis or excessive fluid replacement. Instead, pancreatic pleural effusion is caused by a ruptured pancreatic duct.^2^ Various treatment options are available, but there is currently no consensus on the optimum treatment for mediastinal pancreatic pseudocysts and pancreatic pleural effusion. We herein report a case of a mediastinal pancreatic pseudocyst and pancreatic pleural effusion that was treated with endoscopic transpapillary pancreatic duct drainage.

## CASE REPORT

**FIGURE 1:**
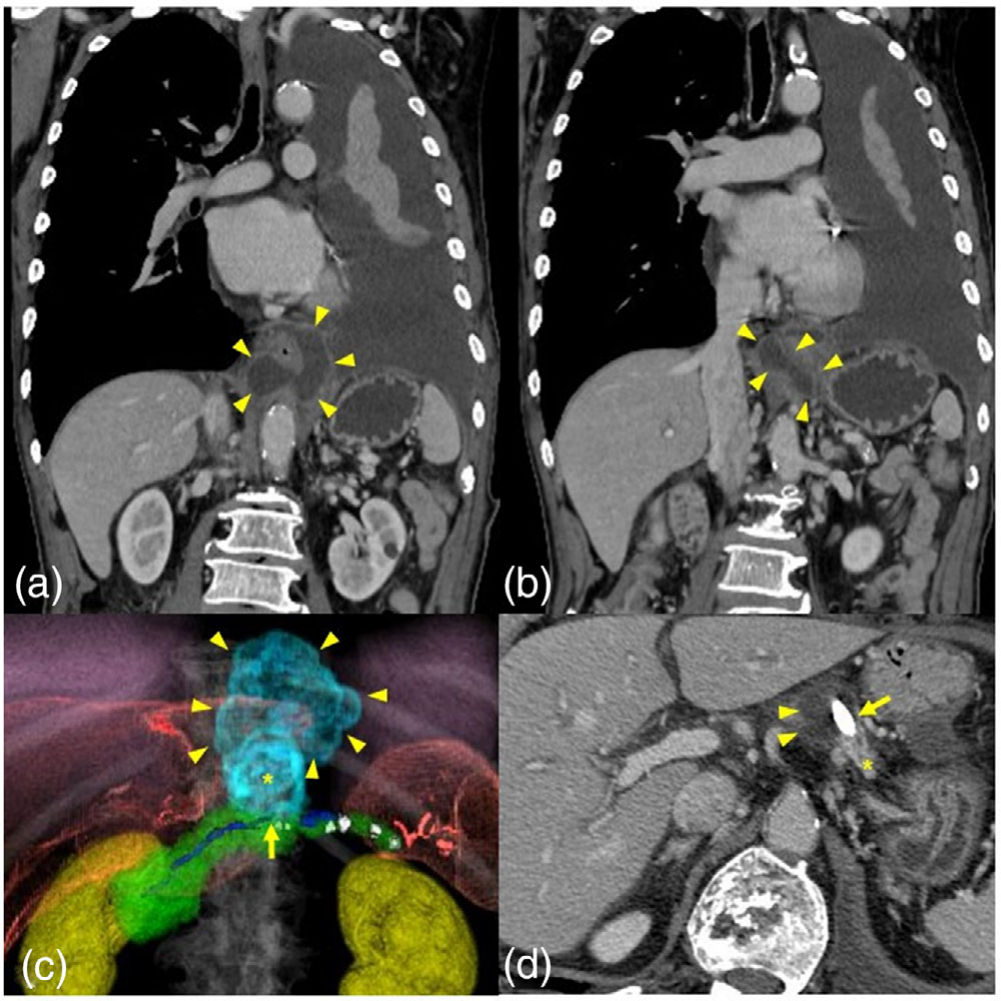
Chest and abdominal computed tomography: (a, b) A pancreatic pseudocyst extended toward the mediastinum and communicated with a mediastinal pseudocyst through the aortic hiatus (yellow arrowhead: pancreatic and mediastinal pseudocyst). (c) Three‐dimensional reconstructed computed tomography showed a connection between the pancreatic pseudocyst and mediastinal pseudocyst (green structure: pancreas, blue line: pancreatic duct, white structure: pancreatic stone, yellow arrowhead: mediastinal pseudocyst, asterisk: pancreatic pseudocyst, yellow arrow: pancreatic duct disruption). (d) The main pancreatic duct had ruptured toward the posterior surface of the pancreas on the proximal side of the pancreatic stone (yellow arrowhead: pancreatic duct rupture, yellow arrow: pancreatic stone, asterisk: pancreas)

**FIGURE 2:**
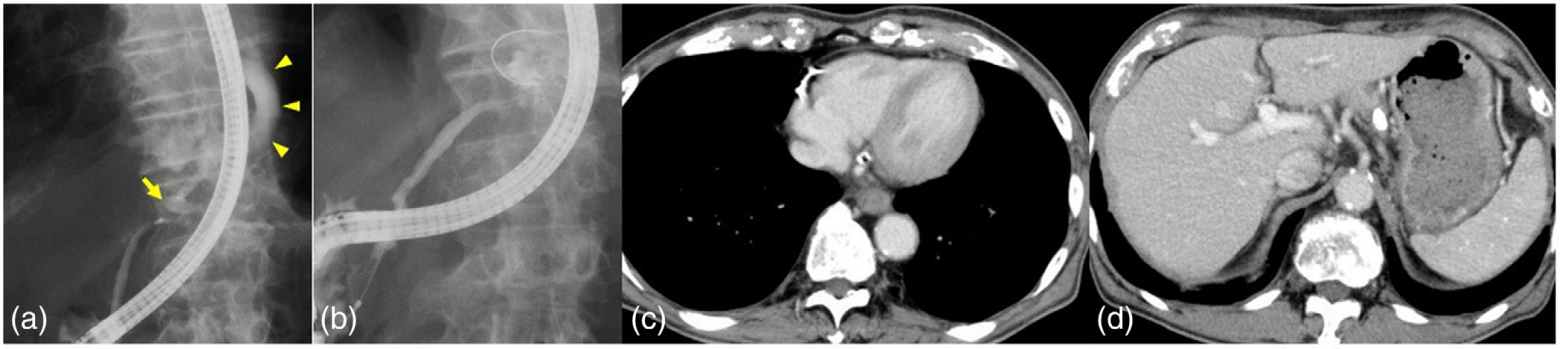
Endoscopic retrograde pancreatography: (a) Leakage of contrast medium was observed from the vicinity of the pancreatic stone to the outside of the pancreas, and a pancreatic pseudocyst and mediastinal pseudocyst were visualized (yellow arrowhead: pseudocyst, yellow arrow: pancreatic stone). (b) A guidewire was inserted into the pancreatic pseudocyst. (c, d) Follow‐up computed tomography after endoscopic transpapillary drainage. The (c) mediastinal pseudocyst and (d) pancreatic pseudocyst had completely disappeared

This case involved an 81‐year‐old man who had been diagnosed with alcoholism and chronic pancreatitis. Twelve and two months previously, he developed dyspnea due to pleural effusion of unknown origin, and a chest tube was inserted for symptomatic treatment at another hospital. He was admitted to our facility with the chief complaint of epigastralgia and dyspnea. On admission, he was given 3 L of oxygen by nasal cannula. He had a fever (37.7°C), tachypnea (25 breaths/min), and tachycardia (105 beats/min). Blood tests revealed a slight inflammatory response and elevated pancreatic enzymes (Table 1). Chest computed tomography (CT) revealed pleural effusion occupying most of the left thorax and posterior mediastinal fluid collection in front of the aorta (Figure 1a,b). Abdominal CT showed fat stranding and fluid collection around the pancreas and multiple pancreatic calcifications in the main pancreatic duct. The patient was diagnosed with acute exacerbation of chronic pancreatitis. The main pancreatic duct had ruptured toward the posterior surface of the pancreas on the proximal side of the pancreatic stone, and a connection with fluid collection around the pancreas was observed (Figure 1d). The fluid collection around the pancreas extended toward the mediastinum and communicated with the mediastinal fluid collection through the aortic hiatus (Figure 1a,b). Furthermore, three‐dimensional reconstruction (SYNAPSE VINCENT; Fujifilm Medical, Tokyo, Japan) showed a connection between a pancreatic pseudocyst and mediastinal pseudocyst (Figure 1c). The mediastinal fluid collection and left pleural effusion were believed to be a mediastinal pancreatic pseudocyst and pancreatic pleural effusion with an extended pancreatic pseudocyst formed by pancreatic duct disruption. A chest tube was placed as in the previous treatment. The pleural effusion analysis demonstrated a significantly elevated amylase level (2820 IU/L), suggesting pancreatic pleural effusion. About 200 ml/day of pleural effusion was drained from the chest tube, and this volume did not decrease; therefore, another drainage technique was considered necessary. Because pancreatic duct rupture was confirmed by CT, we considered that endoscopic transpapillary pancreatic duct drainage was appropriate. We performed endoscopic retrograde pancreatography (ERP), which showed excretion of a protein plug from the duodenal papilla, and a simple X‐ray showed a 16.8 × 8.6‐mm pancreatic stone on the right side of the vertebral body. ERP showed wall irregularity of the main pancreatic duct in the pancreatic head and body. Furthermore, leakage of contrast medium was observed from the vicinity of the pancreatic stone to the outside of the pancreas, and a pancreatic pseudocyst and mediastinal pseudocyst were visualized by ERP (Figure 2a). We succeeded in seeking the pancreatic pseudocyst cavity from the fistula of the pancreatic duct with a 0.025‐inch guidewire (VisiGlide; Olympus, Tokyo, Japan) (Figure 2b). Finally, a 5‐Fr endoscopic nasopancreatic drainage tube (Wilson‐Cook Medical Inc., Winston‐Salem, NC, USA) was placed in the pancreatic pseudocyst. Follow‐up CT 4 days after ENPD tube placement showed the disappearance of the pancreatic pseudocyst, mediastinal pseudocyst, and left pleural effusion (Figure 2c,d). Cytological examination of the pancreatic juice and pseudocyst fluid revealed only inflammatory cells (class II). An internal fistula stent (7‐Fr, 9‐cm Geenen pancreatic stent; Wilson‐Cook Medical) was indwelled to bridge the fistula of the pancreatic duct for continuous drainage. The chest tube was removed, and the patient was discharged on postoperative day 18. Thereafter, CT showed recurrence of the pancreatic pseudocyst 6 months after the initial pancreatic duct stent placement. Obstruction of the pancreatic duct stent occurred, and the pancreatic duct stent was replaced. The pancreatic duct stent was thereafter replaced regularly every 4 months as a preventative. At the time of this writing, 27 months had passed without recurrence.

**TABLE 1:** Blood test results at the time of admission

**Complete blood count**		**Biological examination**	
RBC	400 × 10^4^ /μl	Na	137 mEq/L
Hgb	14.0 g/dl	K	4.1 mEq/L
Plt	25.0 × 10^4^ /μl	Cl	104 mEq/L
WBC	10,800 /μl	Ca	8.8 mg/dl
		Alb	3.0 g/dl
		BUN	19.4 mg/dl
		Cr	0.62 mg/dl
Blood coagulation tests		T‐Bil	1.1 mg/dl
PT	100%	D‐Bil	0.3 mg/dl
PT‐INR	0.94	ALP	141 IU/L
APTT	34.2 seconds	γGTP	63 IU/L
		AST	22 IU/L
		ALT	25 IU/L
		LDH	181 U/L
Tumor markers		Amylase	424 IU/L
CEA	2.4 ng/ml	Lipase	529 IU/L
CA19‐9	7.8 U/ml	CRP	3.16 mg/dl
Span‐1	15.8 U/ml	PCT	1.09 ng/ml
DUPAN‐2	34 U/ml	BNP	16.2 pg/ml

Alb, albumin; ALP, alkaline phosphatase; ALT, alanine aminotransferase; APTT, activated partial thromboplastin time; AST, aspartate aminotransferase; BNP, brain natriuretic peptide; BUN, blood urea nitrogen; Ca, calcium; CA19‐9, carbohydrate antigen; CEA, carcinoembryonic antigen; Cl, chloride; Cr, creatinine; CRP, C‐reactive protein; D‐Bil, direct bilirubin; DUPAN‐2, duke pancreatic monoclonal antigen type 2; γGTP, γ‐glutamyltransferase; Hgb, hemoglobin; K, potassium; LDH, lactate dehydrogenase; Na, sodium; PCT, procalcitonin; Plt, platelets; PT‐INR, prothrombin time–international normalized ratio; PT, prothrombin time; RBC, red blood cells; Span‐1, S‐pancreas‐1 antigen; T‐Bil, total bilirubin; WBC, white blood cells.

## DISCUSSION

Notably, the mediastinal pancreatic pseudocyst and pancreatic pleural effusion, in this case, were treated by endoscopic transpapillary pancreatic duct drainage. The definitive diagnosis was based on imaging studies showing cystic lesions extending from the pancreas into the mediastinum and an elevated pleural effusion amylase level. When performing endoscopic transpapillary drainage, it is important that the connection between the pancreatic/mediastinal pseudocysts and the pancreatic duct is confirmed.^3^ The best way to diagnose a pseudocyst of the pancreas and its mediastinal extension is to perform contrast‐enhanced CT of the chest and abdomen.^3^ Magnetic resonance cholangiopancreatography can be used to evaluate the connection between the main pancreatic duct and the pancreatic pseudocyst. However, this imaging technique was not used in our case because the connection was confirmed by contrast‐enhanced CT and three‐dimensional reconstructed CT showed the connection between the pancreatic pseudocyst and mediastinal pseudocyst. Contrast‐enhanced CT helps to assess the relationship between the pseudocyst itself and the surrounding structure, which plays an important role in treatment planning.^3^, ^4^ In our case, the connection between the pancreatic duct and mediastinal pseudocyst was clearly identified, and we were able to select endoscopic transpapillary pancreatic duct drainage.

Management of mediastinal pancreatic pseudocysts depends on their size, quantity, location, and relationship with adjacent anatomical structures; the presence of infection or hemorrhage; the presence of communication of the cyst with the pancreatic duct; and symptom severity.^5^ Small mediastinal pancreatic pseudocysts may resolve spontaneously, but this is a rare event and requires prolonged conservative management including somatostatin analogues^6^. Surgical treatment has been reported for pancreatic and mediastinal pseudocysts; however, surgery is highly invasive.^7^ Ajmera and Judge^5^ reported a treatment algorithm for mediastinal pancreatic pseudocysts. They suggested that all unstable patients with life‐threatening complications should be treated with open surgery and that the management of stable patients should depend on the size of the pseudocyst and the presence of symptoms. Surgical treatment is especially desirable for pseudocysts with infection, obstruction, rupture, or bleeding.^5^ These signs were not observed in our patient, and we considered that surgical treatment was not indicated. Some reports have described endoscopic ultrasound‐ and CT‐guided pseudocyst drainage.^8^ These are minimally invasive direct drainage methods for mediastinal pseudocysts and may be effective for cases of suspected infection or abscess. However, these techniques only drain the cystic cavity; the pancreatic duct fistula must close over time. Because most mediastinal pancreatic pseudocysts are caused by disruption of the pancreatic duct, we consider that endoscopic transpapillary pancreatic duct drainage is an appropriate treatment for mediastinal pancreatic pseudocysts connected to the pancreatic duct because it allows for simultaneous drainage of the pancreatic duct and pseudocyst. Moreover, endoscopic transpapillary pancreatic duct drainage can prevent the dissemination of inflammatory cells, suppress pancreatic juice leakage, and promote fistula closure. Twelve reports have described mediastinal pseudocysts treated by ERP as the initial drainage technique (Table 2).^1^, ^7^, ^8^, ^9^, ^10^ Of these, only one case could not be controlled by ERP alone. Surgery was performed in this case because it pierced the bronchus and the patient's respiratory condition deteriorated.^9^ Initial drainage in the remaining 11 cases was placed in the main pancreatic duct to bridge the pancreatic duct rupture, and resolution of the pancreatic pseudocyst was achieved. In our case, the endoscopic nasopancreatic drainage tube was directly placed in the pancreatic pseudocyst for transpapillary drainage. As a result, the cyst disappeared earlier than in patients who underwent drainage of the main pancreatic duct. Transpapillary drainage is a potential first‐line treatment for mediastinal pseudocysts with an apparent connection to the pancreatic duct because endoscopic pancreatic stent placement ultimately enables physiological and sustained pancreatic juice drainage, and it can promote cyst drainage and closure of pancreatic duct rupture. Additionally, when transpapillary drainage is performed, the placement of a drainage tube in the pancreatic pseudocyst may facilitate early cyst resolution. However, for patients with a large mediastinal pancreatic pseudocyst or severe infection, treatment options including endoscopic ultrasound‐guided cyst drainage and surgical treatment should be considered. There are no clear criteria for which option should be the first‐line treatment, and it is important to consider each case individually.

**TABLE 2:** Reported cases of transpapillary drainage for mediastinal pancreatic pseudocysts

**No**.	**Reference**	**Age (years), sex**	**Drainage stent**	**Drained area**	**Clinical course**	**Resolution (days)**
1	Mallavarapu et al. (2001)	44, male	7‐Fr plastic stent	MPD	The cyst disappeared and the stent was removed.	Not reported
2	Mallavarapu et al. (2001)	42, female	8.5‐Fr plastic stent	MPD	The cyst disappeared and the stent was removed.	7
3	Kim et al. (2003)	53, male	7‐Fr plastic stent	MPD	The cyst disappeared and the stent was regularly exchanged.	3
4	Musana et al. (2004)	69, male	7‐Fr plastic stent	MPD	Endoscopic stone removal was performed and the stent was removed.	41
5	Thomson and Wigmore (2004)	68, female	5‐Fr plastic stent	MPD	The cyst did not disappear and distal pancreatectomy was performed.	98
6	Bhasin et al. (2005)	28, male	5‐Fr ENPD tube	MPD	The cyst disappeared and the ENPD tube was removed.	28
7	Yasuda et al. (2007)	46, male	Not reported	MPD	The cyst disappeared and distal pancreatectomy was performed for stone removal.	51
8	Rana et al. (2012)	42, male	5‐Fr plastic stent	MPD	The cyst disappeared.	14
9	Rana et al. (2015)	49, male	5‐Fr plastic stent	MPD	The cyst disappeared.	56
10	Hirosawa et al. (2016)	58, male	5‐Fr plastic stent	MPD	The cyst did not disappear and an ENPD tube was placed in the MPD.	At least 19
11	Liao et al. (2022)	57, male	Not reported	MPD	Endoscopic stone removal was performed.	58
12	Inomata et al. (2022)	66, male	5‐Fr ENPD tube	MPD	The cyst disappeared and ESWL and endoscopic stone removal were performed.	Not reported
13	Our case	81, male	5‐Fr ENPD tube	PPC	The cyst disappeared and the ENPD tube was exchanged for a plastic stent.	4

ENPD, endoscopic nasopancreatic drainage; ESWL, extracorporeal shockwave lithotripsy; MPD, main pancreatic duct; PPC, pancreatic pseudocyst.

## CONFLICT OF INTEREST

The authors declare no conflict of interest.

## FUNDING INFORMATION

None.
